# Accuracy and Efficacy of a Patient-Specific Drill Guide Template System for Lumbosacral Junction Fixation in Medium and Small Dogs: Cadaveric Study and Clinical Cases

**DOI:** 10.3389/fvets.2019.00494

**Published:** 2020-01-09

**Authors:** Toru Fujioka, Kohei Nakata, Yukiko Nakano, Yuta Nozue, Taku Sugawara, Naoyuki Konno, Sadatoshi Maeda, Hiroaki Kamishina

**Affiliations:** ^1^The United Graduate School of Veterinary Sciences, Gifu University, Gifu, Japan; ^2^Ivy Animal Clinic, Okayama, Japan; ^3^The Animal Medical Center of Gifu University, Gifu University, Gifu, Japan; ^4^Department of Spinal Surgery, Research Institute for Brain and Blood Vessels-Akita, Akita, Japan; ^5^Konno 3D Design, Akita, Japan; ^6^Center for Highly Advanced Integration of Nano and Life Sciences, Gifu University, Gifu, Japan

**Keywords:** patient-specific, drill guide template, screw placement, lumbosacral junction, dog

## Abstract

**Objectives:** To develop drill guide templates as an intraoperative guide, and to evaluate the accuracy and safety of screw placement in the lumbosacral junction.

**Samples:** Canine cadaveric specimens of the lumbosacral junction (*n* = 6), and clinical cases of lumbosacral instability (*n* = 3).

**Procedures:** Computed tomography data of the lumbosacral region of cadaveric specimens and clinical cases were obtained. The optimum screw trajectories were determined and drill guide templates were fabricated using a three-dimensional printing system. Drill holes were made using the templates in cadaveric specimens and clinical cases, and lumbosacral fixation was performed in clinical cases. Computed tomography images were obtained to compare the planned and postoperative drill hole trajectories, and the accuracy and safety of drilling and screw placement in the lumbosacral junction were evaluated.

**Results:** Thirty-six drill holes were made in cadaveric specimens. The overall mean drill hole deviation was 2.05 ± 1.32 mm. A total of 12 screws were placed in the lumbosacral junctions of three clinical cases. The overall mean drill hole deviation was 2.43 ± 1.09 mm. Clinical signs improved within 2 weeks in the clinical cases. All drill holes were completely located within the bone in cadaveric specimens and clinical cases.

**Conclusion and Clinical Relevance:** The surgical procedures using the drill guide templates were performed safely with good clinical outcomes. The drill guide template system provided useful surgical guidance to safely and precisely perform screw placement for lumbosacral fixation surgery in small dogs.

## Introduction

Lumbosacral luxation in dogs caused by degenerative lumbosacral stenosis (DLSS), trauma (fracture and/or luxation), discospondylitis, neoplasia. DLSS is characterized by stenosis of the spinal canal, causing compression of the cauda equina in dogs. Although this condition is most commonly seen in medium- and large-sized dogs, it can also be seen in small dogs, typically presenting with caudal lumbar back pain (1). Lumbosacral luxation in small animals usually result from trauma such as hitting by car or falling from high place, when external forces overwhelm the physiological stabilizers of spine (2).

Surgical treatment for DLSS is the standard choice of treatment in the presence of severe caudal lumbar pain or neurologic deficits that do not respond to conservative therapies (3, 4). The reported surgical techniques for DLSS include dorsal laminectomy alone or in combination with partial discectomy, dorsal laminectomy combined with fixation and fusion, or lateral foraminotomy (2, 3, 5–8). Although the reported rate of short-term improvement of clinical signs with decompressive surgery is as high as 78–93% (1, 9), the long-term recurrence rate of clinical symptoms is also relatively high at 17–38% (1, 9). Decompressive surgery alone could further aggravate instability of the lumbosacral junction, and dorsal vertebral body fixation should therefore be considered in cases with a high risk of postoperative instability at the surgical site (4, 7, 9).

Fixation of the lumbosacral junction is commonly achieved by implants, such as pins and/or screws, placed within the pedicles or vertebral bodies along with polymethylmethacrylate (PMMA) (5, 10, 11), or by using a pedicle screw-rod fixation system (1, 6, 12, 13). The advantage of dorsal fixation is that it provides stability to the lumbosacral junction and can reduce dynamic compression of the spinal cord (1, 2, 6). However, the placement of implants for lumbosacral junction fixation carries a potential risk of iatrogenic injury to vital structures including cauda equine, nerve roots, and vasculature (3). The accurate placement of implants is thus of upmost importance to achieve rigid fixation and also to avoid injury to the surrounding anatomy. Although the optimal implant trajectory for lumbosacral fixation has been reported (3), freehand insertion of implants is widely performed in specialty between veterinary and practice, which is oftentimes problematic for placing the implants in the ideal trajectory. In addition, the freehand technique carries an increased risk of complications, such as iatrogenic injury of cauda equine, nerve roots, and vasculature, together with insufficient stability due to a lack of rigid attachment to the vertebrae and complete biologically fusion.

We previously reported a patient-specific drill guide template (14), which was developed as an inexpensive device to accurately guide spinal fixation screws in the thoracolumbar vertebrae of dogs. The present study assessed the accuracy of patient-specific drill guide templates for screw placement in the lumbosacral junctions of canine cadavers. We also applied this system to three clinical cases with lumbosacral luxation.

## Materials and Methods

### Cadaveric Study

All procedures were performed in accordance with the guidelines regulating animal use and ethics at Gifu University (approval number: 17211). The caudal lumbar spine (L6-7) and sacrum were removed from 6 cadavers of adult beagles that were used in other research projects and euthanized for reasons unrelated to the present study. The mean age was 3 years (range: 1.8–4.5 years). The median body weight was 13.5 kg (range: 10.7–18 kg). All epaxial muscles were left intact. The spinal specimens were wrapped in saline (0.9% NaCl) solution-soaked paper towels and stored at −20°C until use.

### Determination of Screw Trajectories

**Figure 1:**
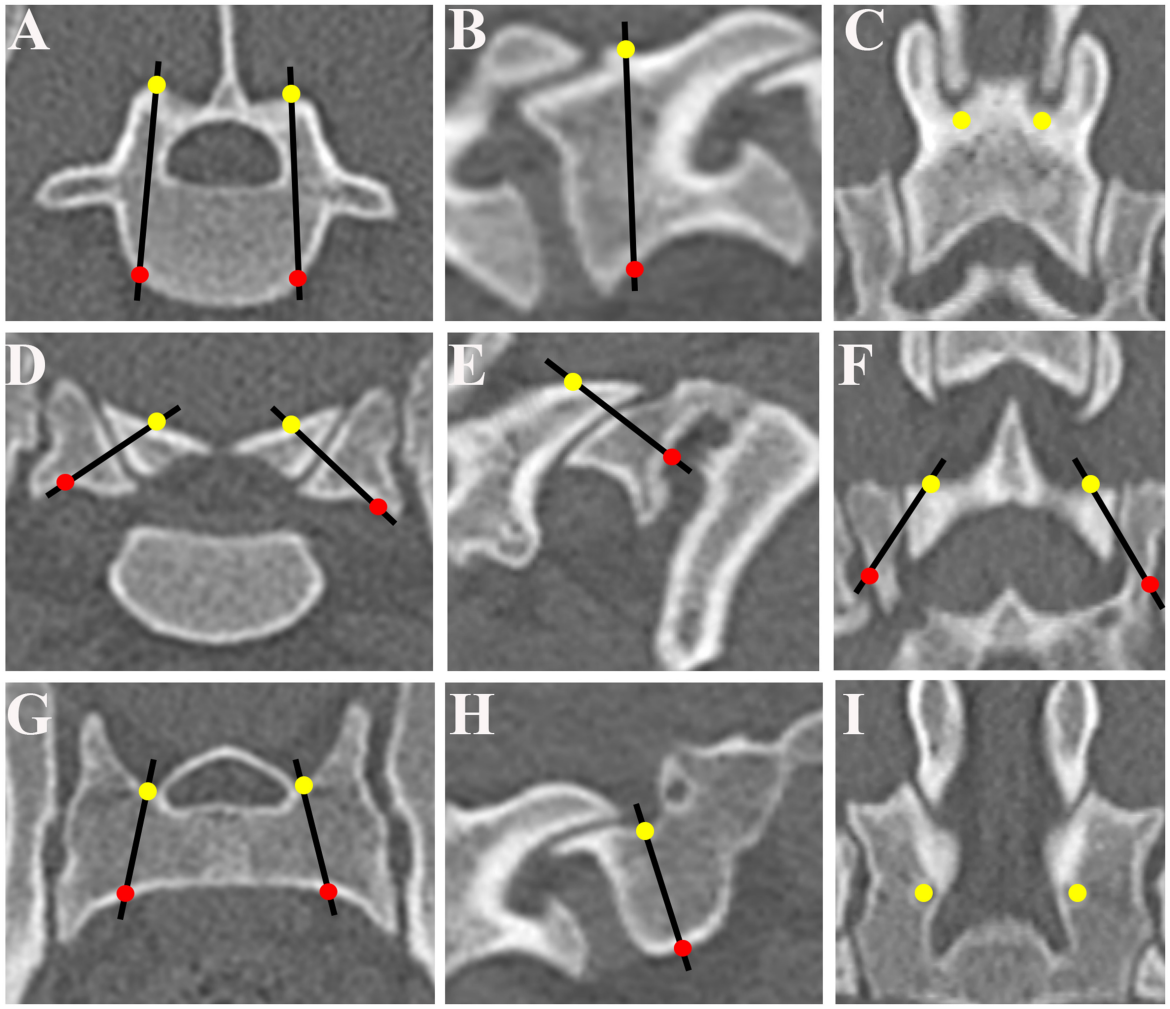
Determination of optimum screw trajectories. The trajectories and the coordinates of the bone entry and exit points were determined and six trajectories were selected for L7 on the transverse plane **(A)**, sagittal plane **(B)**, and dorsal plane **(C)**, for L7-S1 articular processes on the transverse plane **(D)**, sagittal plane **(E)**, and dorsal plane **(F)**, and for the sacrum on the transverse plane **(G)**, sagittal plane **(H)**, and dorsal plane **(I)**. The entry points of the drilling holes are indicated by solid yellow circles and the exit points by solid red circles. The optimum screw trajectories are indicated by black lines.

A computed tomography (CT) scanner (Alexion Advance, Canon Medical System Corporation, Tochigi, Japan) was used to obtain 0.5 mm-slice preoperative images of the caudal lumbar and sacrum region. The images obtained by CT imaging were exported to three-dimensional (3D)/multiplane imaging software (Ziostation, Ziosoft, Tokyo, Japan) in the Digital image processing and Communications in Medicine (DICOM) format. We then selected the optimal implant trajectories for L7, the L7-S articular processes, and the sacrum using multi-planar reconstruction (MPR) images. The entry point of the L7 implant was at the base of each dorsal articular process. In the L7-S articular facet, we planned drill trajectories in order to place the implant through the caudal articular process of L7 and the cranial articular surface of the sacrum. Specifically, the trajectories were planned such that the angle of the screw was 30–45° in the sagittal plane and 45–60° in the transverse plane, extending from the dorsomedial surface of L7 to the ventrolateral surface of the sacrum. The entry point of the sacrum screw was cranial aspect of sacrum, medial to the caudal articulation of L7 and the sacrum. The trajectories were planned so as to avoid injury to surrounding important structures such as the cauda equina, nerve roots, and blood vessels. The 3D coordinates of the entry points and exit points on L7, the articular processes, and the sacrum were subsequently recorded and used to design the drill guide templates (Figure 1).

### Design and Fabrication of Patient-Specific Drill Guide Templates

**Figure 2:**
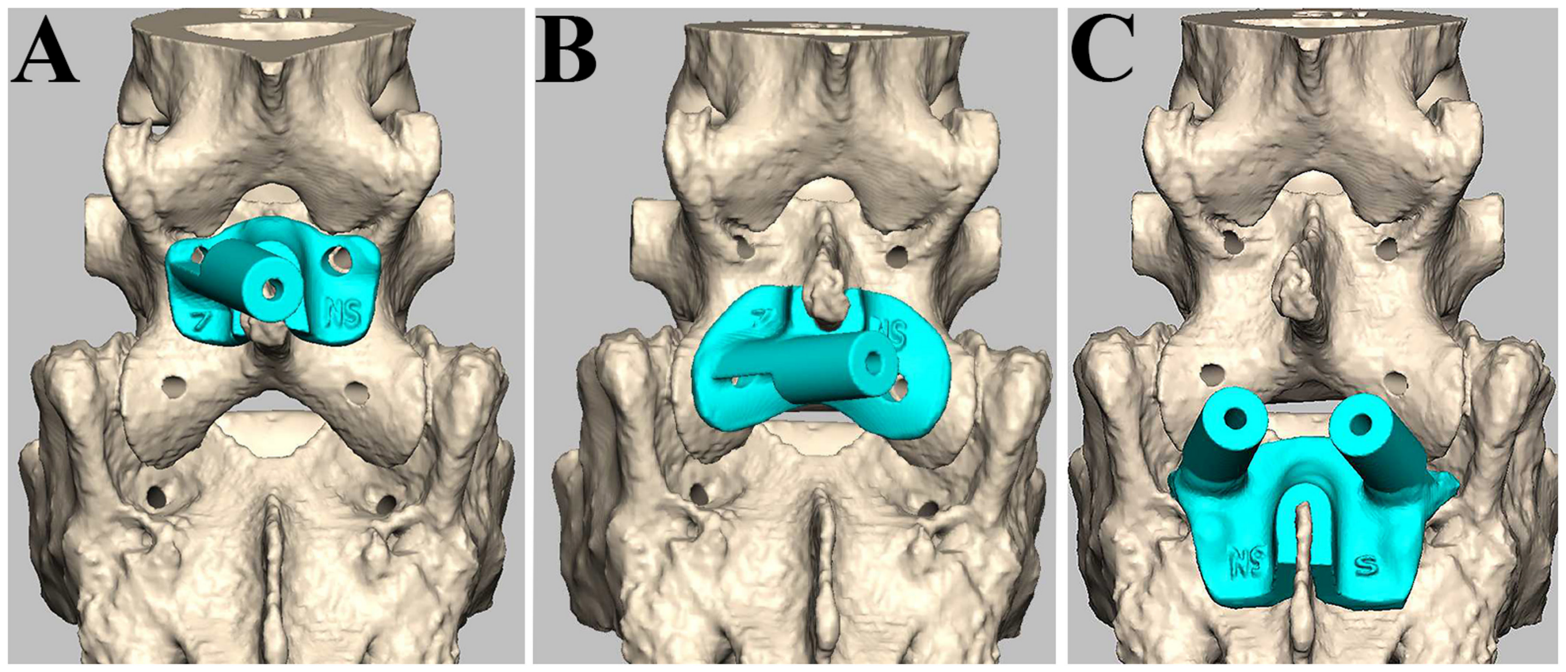
Design of patient-specific drill guide templates. The drill guide template for the left side of L7 **(A)**, the left articular process **(B)**, and both sides of S1 **(C)**.

We designed and fabricated the drill guide templates as we reported previously (14). Briefly, bone processing data were extracted from the DICOM data using image processing software (VG Studio Max, Volume Graphics GmbH, Heidelberg, Germany Mimics, Materialize, Leuven, Belgium) and transferred to 3D modeling software (Freeform, Data Design, Nagoya, Japan) to generate a 3D reconstruction model. Using a 3D printing system (Connex500, Stratasys, Tokyo, Japan), patient-specific drill guide templates were fabricated from non-soluble acryl. The drill guide template consisted of a cylindrical sleeve (inner diameter of 1.5 mm) and a platform to fit the patient-specific 3D shape of the L7 vertebral arch and sacrum (Figure 2). We also made a patient-specific 3D bone model to confirm the fit of the templates and the angle of the drill sleeve in relation to the vertebral body before surgery. The drill guide templates and bone models were sterilized with ethylene oxide gas before surgery.

### Drilling of Cadaveric Vertebrae Using Drill Guide Templates

**Figure 3:**
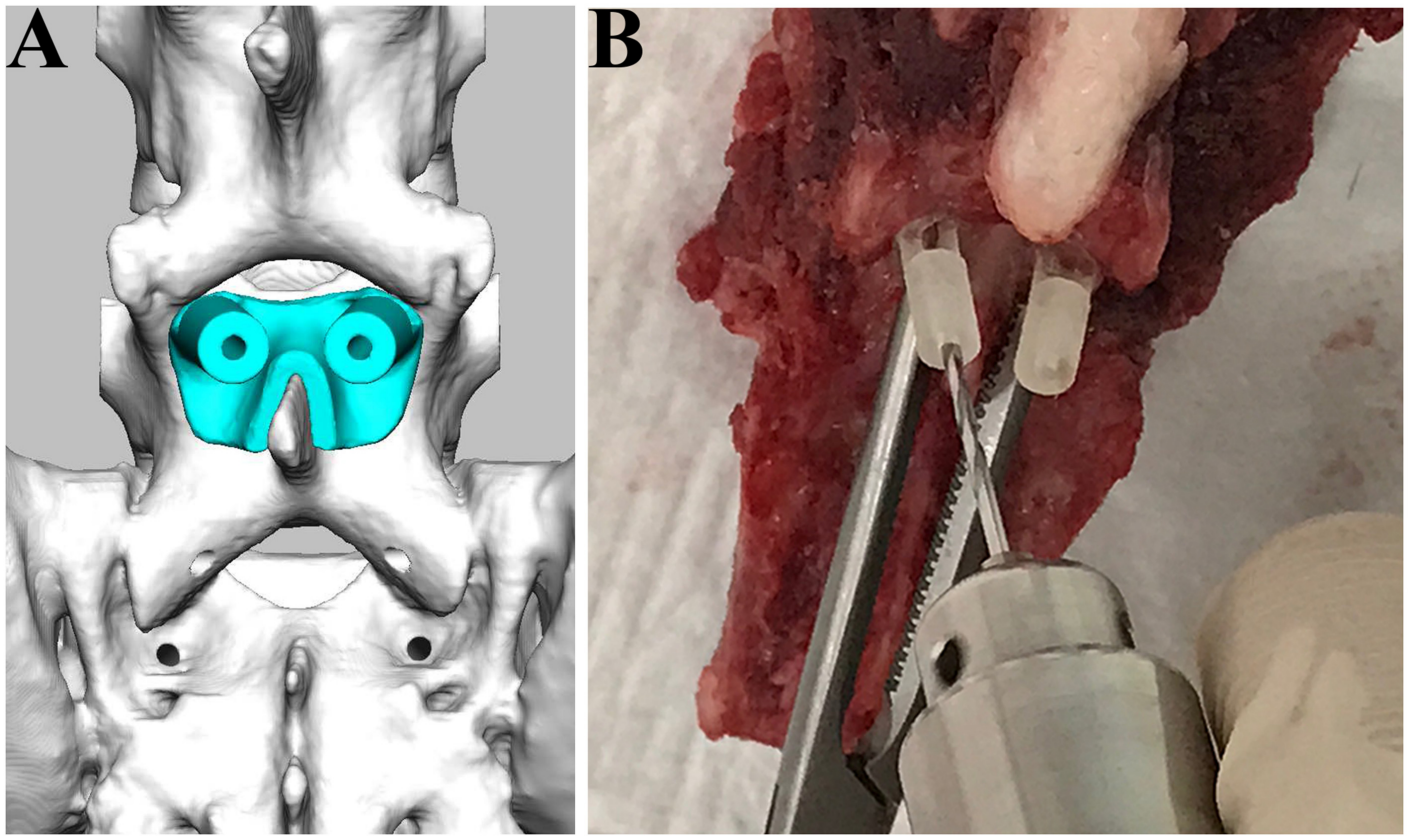
Drilling procedure for L7 using drill guide templates. The drill guide template for L7 was designed **(A)** and applied for the cadaveric spine **(B)**.

Before drilling, the specimens of the caudal lumbar spine (L6-7) and sacrum were thawed in warm water. The spinous process and vertebral arch were exposed by dissecting paraxial muscle tissues from the specimens. Drill guide templates were firmly attached to the lamina by curved Halsted mosquito forceps, and bi-cortical screw holes were made with a drill bit (1.5 mm) (Figure 3). The locations of the drill holes were examined on postoperative CT images.

### Evaluation of the Accuracy and Safety of Drill Guide Templates

**Figure 4:**
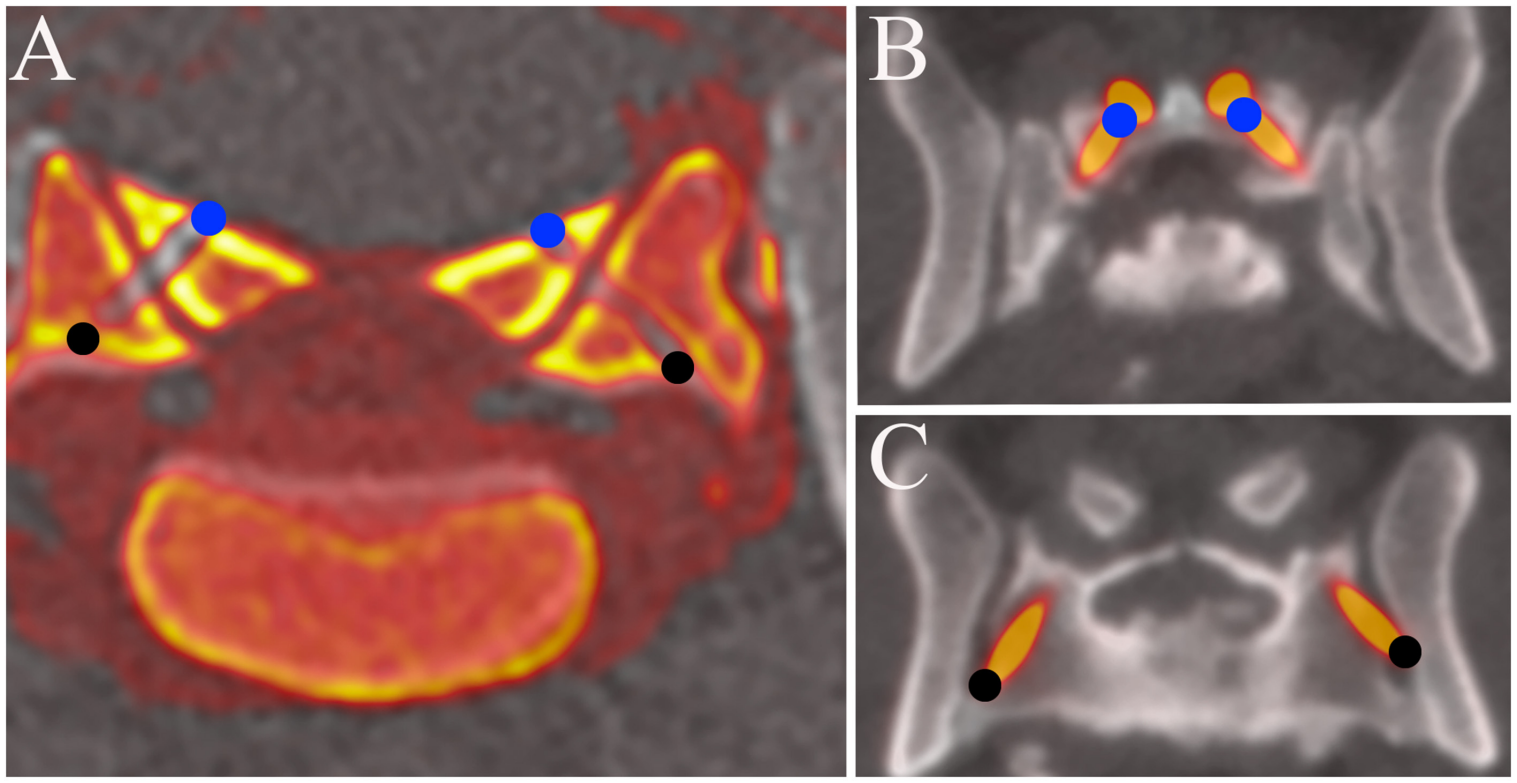
Fusion images of pre and post operative MPR images of the L7-S1 articular processes. **(A)** Cadaveric study. **(B,C)** Clinical case (Case 1). The coordinates of the entry and exit points obtained with the fusion image were compared with those planned before surgery. The entry points of the drill holes or screws are indicated by solid blue circles and the exit points by solid black circles.

To estimate deviations of the screw trajectories, we made fusion images of the preoperative and postoperative CT images. DICOM data were imported into 3D/multiplane imaging software (Ziostation, Ziosoft, Tokyo, Japan), and six landmarks were registered on the postoperative MPR images. The landmarks used were the spinous process, transverse process, and intervertebral foramina for L7, and the intermediate sacral crest, the median sacral crest, and dorsal sacral foramina for the sacrum. Subsequently, fusion images were made by superimposing the postoperative MPR image on the preoperative MPR image, and the coordinates of the entry and exit points of the drill holes on the fusion images were recorded (Figure 4). Deviations in the coordinates of the entry and exit points in each dimension (x, y, z) were evaluated, as in our previous report (14). To evaluate the safety of the drill guide templates, drill hole displacement was graded using postoperative transverse and sagittal CT images as a reference. Grading of the drill hole trajectories was as follows: Grade 0 (containing)—the drill hole trajectory was completely within the wall of the bone structure; Grade 1 (exposure)—the drill hole trajectory invaded the wall of the bone structure, while more than 50% of the drill hole diameter remained within the bone; Grade 2 (perforation)—the drill hole trajectory invaded the bone structure and more than 50% of the drill hole diameter was outside the bone structure; Grade 3 (penetration)—the drill hole trajectory completely penetrated outside the bone structure (15–17).

### Clinical Cases

Imaging studies and surgical procedures were performed at the Animal Medical Center of Gifu University with the informed consent of the owners. We performed surgical stabilization using drill guide templates in three clinical cases. We performed neurological examinations, CT imaging, and MRI (0.4-Tesla APERTO Eterna, Hitachi Healthcare, Tokyo, Japan). The neurological status of each dog was classified using a modified Frankel score (MFS). All three cases were diagnosed as luxation of the lumbosacral junction. One case showed chronic progressive paraparesis and ataxia, which were thought to result from spinal instability based on imaging findings of degenerative changes, such as intervertebral disc degeneration, and spondylosis deformans (2, 9, 18–20). The other two cases were diagnosed as spinal instability due to trauma, based on imaging findings of lumbosacral luxation. Clinical information and imaging findings of the three cases are summarized in Table 1.

**Table 1:** Clinical information and findings of clinical cases.

**Case no**.	**Breed**	**Body weight (kg)**	**Age (years)**	**Sex**	**Clinical sign**	**Diagnosis**	**Surgery**	**Screw location**	**Follow-up period (months)**	**Neurological grade**		
										**Pre-surgery**	**Perioperative follow-up period**	**Last follow-up period**
1	Toy poodle	4.08	8	SF	Paraparesis, caudal lumbar pain, pelvic limb lameness	Lumbosacral subluxation, discospondylitis	Dorsal laminectomy, partial disectomy, transarticular facet fixation	L7-S1	12	3a	4	4
2	Yorkshire terrier	2.9	7.7	F	Caudal lumbar pain	Lumbosacral luxation	L7-S1 screw-PMMA fixation	L7-S1	7	3a	4	4
2	Toy poodle	4.18	7.4	M	Caudal lumbar pain	Lumbosacral luxation	L7-S1 screw-PMMA fixation	L7-S1	1	5	5	5

*Neurological grades were defined based on Modified Frankel Score: 5, Normal gait with paraspinal hyperesthesia; 4, Ambulatory paraparesis; 3a, Non-ambulatory paraparesis (ability to bear weight on the pelvic limbs without support); 3b, Non-ambulatory paraparesis (inability to bear weight on the pelvic limbs without support); 2, Paraplegia with intact nociception; 1, Paraplegia with absent superficial nociception; 0, Paraplegia with absent deep nociception. SF, spayed female; F, intact female; M, intact male*.

### Surgeries

#### Case 1

**Figure 5:**
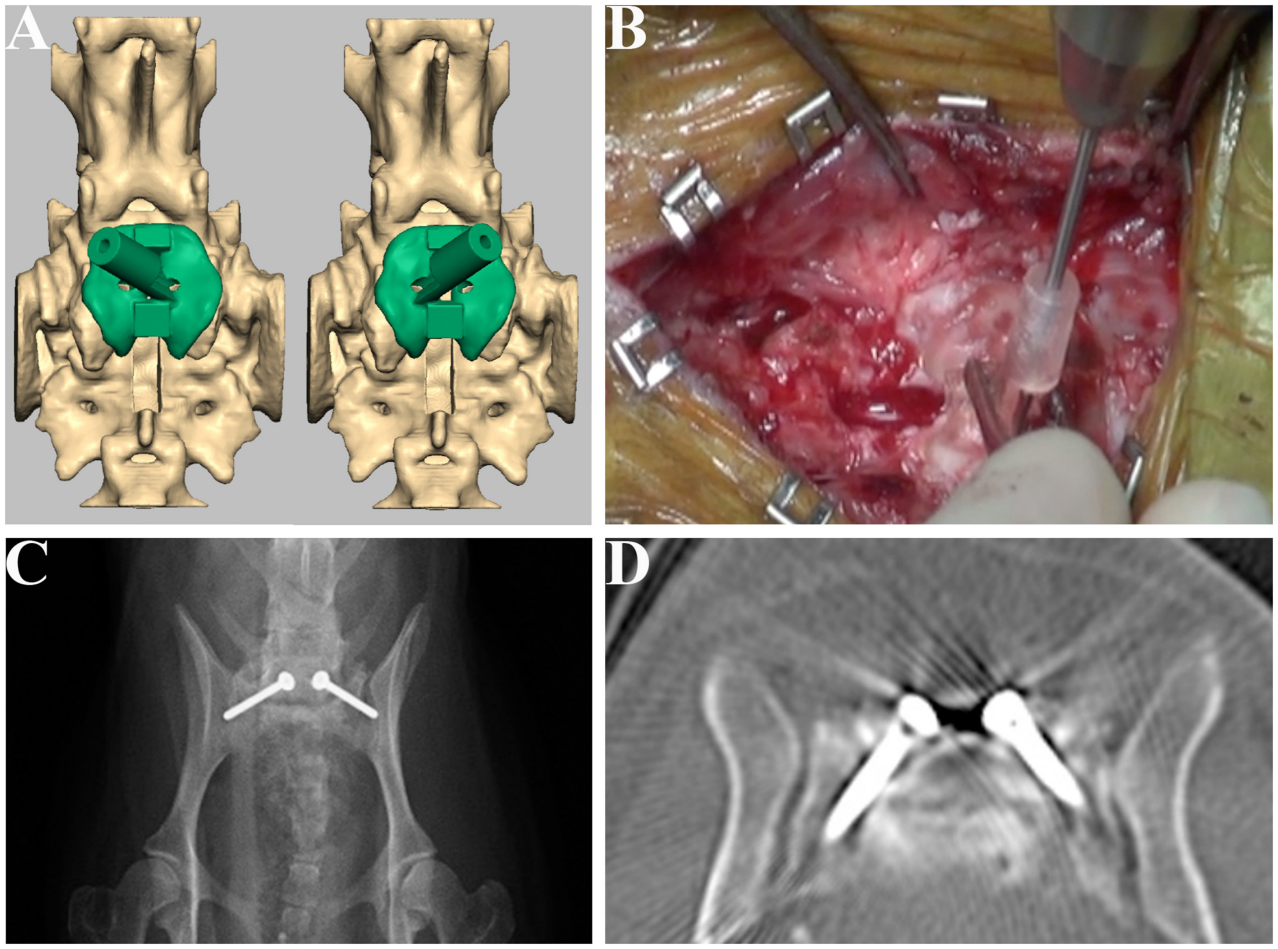
Design of the drill guide templates and placement of the screws for Case 1. Drill guide templates were designed for the articular facet **(A)** and were used at the drilling step **(B)**. Postoperative radiograph **(C)** and CT image **(D)** of the lumbosacral junction.

Based on clinical finding and advanced imaging, dorsal laminectomy with partial discectomy was performed to decompress the cauda equina. After releasing the epaxial musculature from the spinous processes, articular processes, lamina arch of L7, and sacrum, the drill guide template was firmly attached to the caudal articular processes with Adson tweezers. A 0.9-mm pin (0.035″ miniature half-pin, IMEX Veterinary, Inc., Texas, USA) was then inserted into the cylinder sleeve and a drill hole was made while visually confirming the entry point from the opening of the drill sleeve. Subsequently, the depth of the drill hole was measured using a depth gauge, the length of the drill hole was evaluated, and bilateral transarticular facet screws (M 2.0 cortical bone screw, Mizuho, Tokyo, Japan) with a diameter of 2 mm were placed. Postoperative CT imaging was performed (Figure 5).

#### Case 2

After skin incision, the epaxial musculature was released from the vertebral body, and the luxation of the lumbosacral junction was reduced by pulling the spinous process of L7 and the median sacral crest with bone holding forceps or towel forceps while confirming the alignment of the L7-S under fluoroscopy. Screws were placed using the drill guide template; two screws were placed in each of the L7, L7-S articular facets, and sacrum. The head of the screw was exposed 10 mm from the lamina surface for bone cement engagement. Bone cement (Surgical Simplex P, Stryker, Tokyo, Japan) was applied in order to cover the screw heads. The locations of the screws were examined on postoperative CT images.

#### Case 3

**Figure 6:**
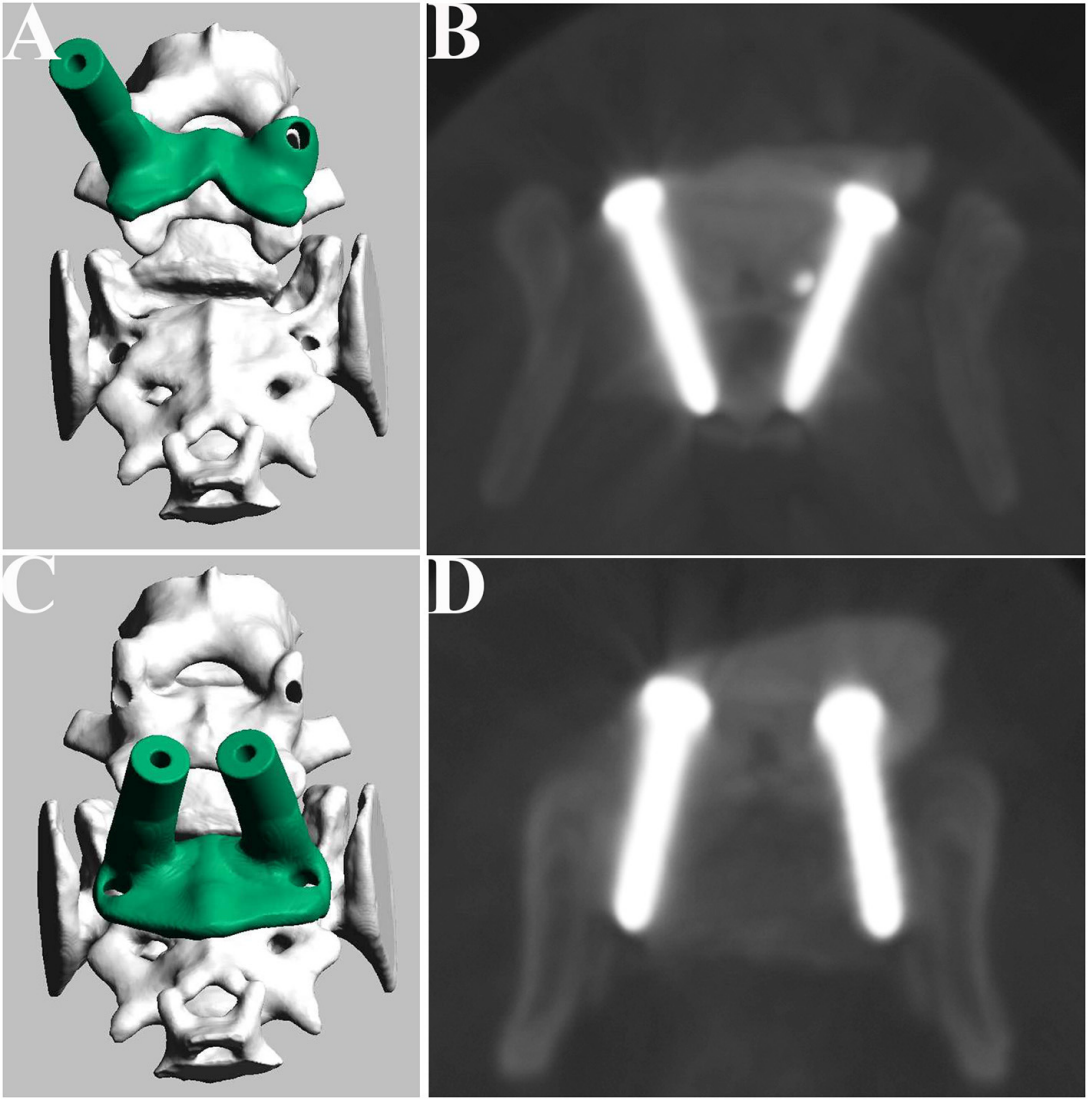
Design of the drill guide templates and placement of the screws for Case 2. Drill guide templates were designed for L7 **(A)** and sacrum **(C)**. Postoperative CT image of the L7 **(B)** and sacrum **(D)**.

A dorsal median incision was performed from the spinous process of L6 to the sacrum. After the epaxial musculature was released from the vertebrae, a thickened ligamentum flavum was observed between the L7 and sacrum, which was removed with dissecting forceps. The luxation of the lumbosacral junction was reduced by pulling the spinous process of L6 to the sacrum with small serrated bone holding forceps or Backhaus towel forceps. After the drill guide template was attached to the L7 and sacrum, a 0.9-mm pin was then inserted into the cylinder sleeve. After inserting the pin, CT imaging was performed to confirm the drilled trajectory, the pin was removed, tapping of the drill hole was performed, and two screws were placed in each of the L7 with the base of each dorsal articular process and sacrum. After the placement of screws, dorsal laminectomy was performed, and bone cement was applied in the same manner as for Case 2. The locations of the screws were examined on postoperative CT images (Figure 6).

### Follow-Up Evaluation

Postoperative (2 weeks) follow-up evaluations consisted of physical and neurological examinations. Neurological grading was only performed when dogs were assessed directly by veterinarians. We also confirmed the clinical progress at 1 year after surgery by telephone interview or physical examination.

### Statistical Analysis

For each vertebra, deviations in millimeters between the planned coordinates and the actual coordinates were obtained. The differences between the mean deviations of the entry points and those of the exit points were statistically compared for each dimension (x, y, z) using statistical software (JMP, SAS institute Japan, Inc., Tokyo, Japan). Data were presented as the mean ± standard deviation. A paired *t*-test was used for the analysis, with significance set at *p* < 0.05.

## Results

### Cadaveric Study

Thirty-six drill holes were made in the cadaveric spinal specimens. In comparison to the planned trajectory, the mean drill hole deviations of the drilled trajectory at the entry and exit points were 1.52 ± 0.77 and 2.57 ± 1.53 mm, respectively. The mean deviations of the exit points were significantly larger than those of the entry points (*p* < 0.05). We also compared the planned trajectory and the drilled trajectory for the mean drill hole deviations of each vertebra between the entry points and exit points in each dimension (x, y, z) (Table 2). The mean deviations of the exit points were significantly larger than those of the entry points on the x axis (mediolateral direction) (*p* < 0.05). In the safety evaluation, we made 12 pedicle screws in L7 and 12 drill holes for transarticular screws. In addition, 12 screw holes were made in the dorsal aspect of the sacrum, medial to the cranial articular surface of the sacrum in a pedicle screw fashion. Exposure of the drill holes from the cortical bone was not observed, and all drill holes were completely contained within the bone (i.e., Grade 0).

**Table 2:** Accuracy of screw locations with drill guide templates for cadaveric spines and clinical cases.

**Trajectory**		**Coordinate of entry point**			**Coordinate of exit point**		
		**x**	**y**	**z**	**x**	**y**	**Z**
Cadaveric spine	Planned	6.76 ± 9.03	9.99 ± 23.36	660.88 ± 171.27	6.2 ± 16.03	−0.32 ± 24.31	658.85 ± 171.83
	Drilled	6.88 ± 9.27	10.11 ± 23.23	660.47 ± 171.17	6.70 ± 15.88	−0.12 ± 24.8	659.10 ± 171.62
	*P*-value	0.31	0.38	0.06	0.03^*^	0.27	0.54
Clinical case	Planned	6.74 ± 7.00	21.35 ± 10.27	894.83 ± 58.09	5.98 ± 9.27	10.87 ± 9.07	893.66 ± 58.83
	Drilled	6.88 ± 7.33	21.28 ± 10.12	894.47 ± 58.04	6.01 ± 9.80	10.86 ± 8.79	893.05 ± 57.59
	*P*-value	0.61	0.80	0.50	0.93	0.98	0.40

*Comparisons of the mean drill hole deviations of cadaveric spinal specimens and clinical cases for the entry and exit points in each dimension (x, y, z)*.

* *p < 0.05*.

### Clinical Cases

A total of 12 screws (4 pedicle screws in L7, 4 screws placed in the dorsal aspect of sacrum and 4 transarticular screws) were placed in the L7, L7-S articular facet, and the sacrum in three clinical cases. The accuracy of the screw locations in clinical cases was evaluated in the same manner as for the cadaveric spinal specimens. The mean drill hole deviations at the entry and exit points were 1.95 ± 0.93 and 2.91 ± 1.01 mm, respectively. The mean deviations of the exit points were significantly larger than those of the entry points (*p* < 0.05). There were no significant differences in the mean screw deviations between the entry and exit points in each dimension (x, y, z) (Table 2). In the safety evaluation, we made 4 pedicle screws in L7, 4 screws placed in the dorsal aspect of sacrum and 4 transarticular screws. Exposure of the screws from the cortical bone was not observed, and all drill holes were completely contained within the bone (i.e., Grade 0). After surgery, the motor function of clinical cases recovered and back pain was resolved within 2 weeks. For case 2, we confirmed by telephone interview that the clinical status was good for more than 1 year after surgery. The results of the follow-up evaluations of the three cases are summarized in Table 1.

## Discussion

In the present study, we evaluated the accuracy and safety of the drill guide template system in cadaveric spinal specimens and clinical cases. The main finding of the present study was that cortical bone was not exposed, as all drill holes were completely located within the bone in both cadaveric spinal specimens and clinical cases. The drill guide template system allowed safe placement of 12 pedicle screws in L7, 12 screw holes in the dorsal aspect of the sacrum, and 12 drill holes for transarticular screws in the cadaveric study, and 4 pedicle screws in L7, 4 screws placed in the dorsal aspect of sacrum, and 4 transarticular screws in clinical cases. We designed and fabricated the drill guide templates, using preoperative CT data, to avoid penetration of the screws to the spinal canal. We encountered no serious complications during surgery or perioperative follow-up period in the three clinical cases. These results corroborate the safety of our patient-specific drill guide templates designed for lumbosacral fixation surgery.

The mean deviations of the exit points were significantly larger than those of the entry points in the cadaveric spine specimens and clinical cases. Additionally, the mean deviations of the exit points were significantly larger than those of the entry points in the x- (mediolateral) direction in the cadaveric study. The cause of this deviation of the drilled holes was thought to be that the angle of the drill sleeve of the drill guide template or the position of the platform changed due to the load in the mediolateral direction during the drilling procedure. We thus need to consider countermeasures for drill misdirection when using our drill guide template system. In order to reduce displacement of the implant, a three-step screw guide template system has been developed for human spinal surgery (15, 21). This system has been verified for accurate implant implantation and shows favorable clinical outcomes in small dogs. We think that it is feasible to further reduce deviation with reference to this system. 3D shape of the drill guide templates ensures that the procedure cannot be affected by spinal alignment changes, such as torsion during drilling and screw placement. In addition, we need to optimize the material of the drill guide template system to reduce deviation. A flexible material that fits tightly to the contour of the vertebral arch is suitable for the lamina cover, while a firm material that resists the load during drilling may be more suitable for the drill sleeve. We think about the material of the drill guide template as follows. The lamina cover of drill guide template is fabricated of a flexible silicone material that fits tightly the vertebral arch, and the drill sleeve should be fabricated of a hard acrylic material that is resistant to loads. Although the deviation between the preoperative planned trajectories and drilled trajectories in this study was larger than that of the thoracolumbar vertebrae in our previous study (14), all drill holes were completely located within the bone, and no intraoperative complications were experienced in the clinical cases.

Limitations of the present study include the small number of clinical cases, short follow-up periods of clinical cases, lack a control group, and the lack of an imaging study at follow-up. Regarding the placement of the implants in the lumbosacral vertebrae, there has been no report on the percentages of cortical perforation by freehand pedicle screw insertion in veterinary medicine, and we were thus unable to compare the accuracy of our drill guide template system with that of freehand insertion and the incidence of complications between these techniques. Therefore, when assessing the accuracy of the drill guide templates, we created surgical fusion images by superimposing the postoperative MPR image on the preoperative MPR image. The stainless-steel screws may have caused metal artifacts on postoperative CT scans, and this may have resulted in incorrect measurement of the screw positions in the clinical cases. Although two-dimensional analysis was performed on the displacement of screw locations, we would like to analyze the accuracy of the drill guide template in a 3D study in the future.

## Conclusion

Based on the evaluation of accuracy and safety, our new drill guide template system was useful for accurate intraoperative screw positioning in lumbosacral fixation surgery for small dogs.

## Disclosure

The drill guide templates were designed and fabricated by NK at Konno 3D design. The drill guide templates are not currently commercially available and are only used at the authors' institution. Preliminary results were presented at the 8th Annual congress of the Asian Society of Veterinary Surgery, Taichung, Taiwan, 1–2 December 2018.

## Data Availability Statement

All datasets generated for this study are included in the article/supplementary material.

## Ethics Statement

The animal study was reviewed and approved by Committee for Animal Research and Welfare of Gifu University. Written informed consent was obtained from the owners for the participation of their animals in this study.

## Author Contributions

TF, TS, and HK: conception and design. TF, YNa, and YNo: acquisition of data. TF, KN, SM, and HK: analysis and interpretation of data. TF: drafting the article and approval of the final version of the manuscript on behalf of all authors. TF and KN: statistical analysis. NK, YNa, YNo, and HK: administrative, technical, and material support. TS, SM, and HK: study supervision. All authors: revision of submitted version of manuscript.

### Conflict of Interest

TF is the founder and current CEO of company Ivy Animal Clinic. NK is a representative of company Konno 3D Design. The remaining authors declare that the research was conducted in the absence of any commercial or financial relationships that could be construed as a potential conflict of interest.
